# Supplementary material for: Effects of Cinnamon Essential Oil on Oxidative Damage and Outer Membrane Protein Genes of Salmonella enteritidis Cells
Source: Foods. 2022 Jul 27;11(15):2234. doi: 10.3390/foods11152234 (PMC9368406; doi:10.3390/foods11152234)
Supplement: Supplementary file 1 [file foods-11-02234-s001.zip › foods-1806897-supplementary.pdf]

Table S1. The primer sequences for q-PCR amplification.

primer	Primer sequence（5'-3')	Fragment length（bp）	Annealing temperature（°C）
16S rRNA-S	AGGTGTAGCGGTGAAATGCG	141	60
16S rRNA-A	CATCGTTTACGGCGTGGACT		60
OmpA-S	GGCTTTGAAATGGGCTACGACT	219	60
OmpA-A	GCCGGTGTCGTGGTCTTTAGT		60
OmpF-S	CCTACTTCTCCGGCGAAACCT	139	60
OmpF-A	CCCTGATACTGGATACCGAAAGAG		60
OmpW-S	CAGCAAAGTGCGTCCTTATGTC	292	60
OmpW-A	ACGATAGCCTGCCGAGAACATA		60
OmpX-S	CGGGCGATTACAACAAAGGTC	248	60
OmpX-A	GTCAACGCTACGAATACGGCTC		60
